# Dual-task improvement of older adults after treadmill walking combined with blood flow restriction of low occlusion pressure: the effect on the heart–brain axis

**DOI:** 10.1186/s12984-024-01412-y

**Published:** 2024-07-12

**Authors:** Yi-Ching Chen, I-Ping Lo, Yi-Ying Tsai, Chen-Guang Zhao, Ing-Shiou Hwang

**Affiliations:** 1 Department of Physical Therapy, College of Medical Science and Technology, Chung Shan Medical University, Taichung City, Taiwan; 2 Physical Therapy Room, Chung Shan Medical University Hospital, Taichung City, Taiwan; 3 Department of Physical Therapy, College of Medicine, National Cheng Kung University, Tainan City, 701 Taiwan; 4 Institute of Allied Health Sciences, College of Medicine, National Cheng Kung University, Tainan City, Taiwan

**Keywords:** Blood flow restriction, EEG, Functional connectivity, Heart rate variability, Dual-task

## Abstract

**Objective:**

This study explored the impact of one session of low-pressure leg blood flow restriction (BFR) during treadmill walking on dual-task performance in older adults using the neurovisceral integration model framework.

**Methods:**

Twenty-seven older adults participated in 20-min treadmill sessions, either with BFR (100 mmHg cuff pressure on both thighs) or without it (NBFR). Dual-task performance, measured through light-pod tapping while standing on foam, and heart rate variability during treadmill walking were compared.

**Results:**

Following BFR treadmill walking, the reaction time (*p* = 0.002) and sway area (*p* = 0.012) of the posture dual-task were significantly reduced. Participants exhibited a lower mean heart rate (*p* < 0.001) and higher heart rate variability (*p* = 0.038) during BFR treadmill walking. Notably, BFR also led to band-specific reductions in regional brain activities (theta, alpha, and beta bands, *p* < 0.05). The topology of the EEG network in the theta and alpha bands became more star-like in the post-test after BFR treadmill walking (*p* < 0.005).

**Conclusion:**

BFR treadmill walking improves dual-task performance in older adults via vagally-mediated network integration with superior neural economy. This approach has the potential to prevent age-related falls by promoting cognitive reserves.

## Introduction

Adults aged 65 and above are subject to fall accidents. In addition to a reduction in muscle strength, fall accidents is related to cognitive decline due to loss of frontal integrity with aging [4]. The 'frontal aging hypothesis' predicts age-related impairments in attentional resource allocation and information processing speed [50], which impact executive functions for multitasking [29]. Due to frontal degeneration, it becomes challenging for older adults to flexibly shift attention between two concurrent tasks [34]. The loss of cognitive resilience partly contributes to age-related falls, particularly when they occur during dual-task scenarios [2, 49].

Blood flow restriction (BFR) is a training method originally used to stimulate muscular development under local hypoxia. It involves applying pneumatic tourniquets to impede venous outflow in the working musculature. The strength gain associated with combined BFR and low-load exercise results from the activation of protein synthesis signaling for enhanced mechanical tension and metabolic stress [38]. Despite its minimal exercise intensity, walking with BFR can augment muscle strength in the elderly [1]. BFR can also affect metabolic cost and cardiovascular responses [31]. It can induce vasoconstriction in the restricted muscles while causing vasodilation in non-restricted areas due to parasympathetic system activation and endothelial nitric oxide (NO) release [21]. The overall impact of BFR on cardiovascular responses is interactively influenced by factors such as occlusion pressure, exercise protocol (resistance vs. aerobic), and application mode (continuous vs. intermittent) [6].

To date, only a few studies have focused on the improvement of frontal executive function by the application of BFR. One study observed that patients with dementia who underwent 6 months of bilateral upper limb compression followed by reperfusion showed improvements in tests of attention and executive function [22]. In healthy older adults, an 8-week dual-task walking program with BFR (occlusion pressure up to 200 mmHg, 20 min/session, 3 sessions/week) resulted in greater improvements in Mini-Mental State Examination scores and increased levels of brain-derived neurotrophic factor (BNDF), compared to those of a control group that did not receive such training [25]. Interestingly, Sugimoto et al. [48] even demonstrated an immediate effect of 15-min BFR treadmill walking (occlusion pressure: 200 mmHg) on the color-word Stroop task, independent of the effect of BFR alone or walking alone. However, it remains uncertain whether a single bout of combined BFR with relatively low occlusion pressure and aerobic exercise can enhance the posture dual-task of older adults with superior neural efficiency. Answering this question is of clinical significance. Lower occlusion pressure (40% systolic artery pressure) has been shown to increase muscle strength without causing elevated blood pressure. Therefore, combining BFR of lower occlusion pressure with aerobic exercise may contribute to fall prevention in older adults by jointly addressing both age-related declines in cognitive function and muscle strength while minimizing the cardiac cost and sympathetic activity.

Supporting the neural connections between the prefrontal cortex, the central autonomic network, and the vagus nerve system [26], higher cardiac vagal activity is linked to superior executive functioning [45]. Within the context of the heart–brain axis, it is possible that dual-task performance can be improved through BFR-related regulation of the autonomic nervous system, which contributes to enhanced executive function and cognitive flexibility. The aim of this study was to compare the acute effects of treadmill walking with and without BFR of low occlusion pressure on posture dual-task performance in older adults, with a special focus on variations in heart rate and EEG characteristics. For older adults, we hypothesized the following: (1) treadmill walking with leg BFR of low occlusion pressure would lead to better performance on a posture dual-task compared to treadmill walking without leg BFR; and (2) the BFR-related organization of the HR kinetics, power spectra of local EEG, inter-regional EEG connectivity, and network topology in various sub-bands would differ from those observed during non-BFR treadmill walking. Scalp EEG of the theta (4–7 Hz), alpha (8–12 Hz), and beta (13–35 Hz) bands were targeted, as they link characteristically to cognitive workload during a posture dual-task in older adults [36].

## Methods

### Subjects

A total of twenty-seven older adults (13 males and 14 females), with an average age of 68.6 ± 4.1 years participated in this study. None of participants reported having known neuromuscular conditions or degenerative neurological diseases that could affect locomotion. These older adults were community-dwelling and did not have significant impairments in walking capacity. They had a regular habit of exercising for at least 1 h daily. The study was approved by the Institutional Review Board of the National Cheng Kung University Medical College Hospital (No. B-ER-110-530). In accordance with the Declaration of Helsinki, all participants provided informed consent to participate prior to the experiment.

### Experimental procedures

**Fig. 1:**
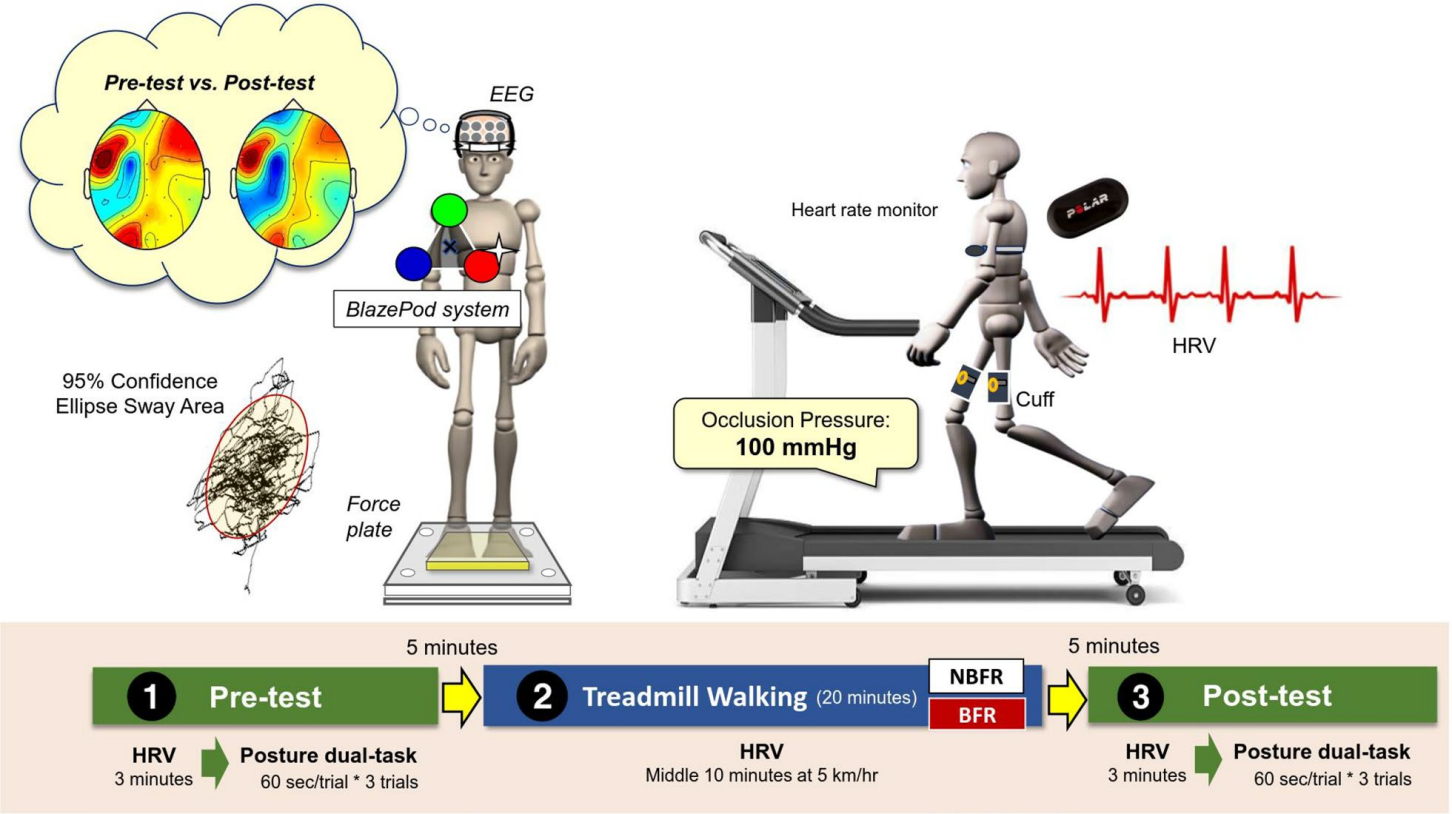
A schematic illustration of the system setup, physiological measures, and flowchart of the experimental process. The pre-test and post-test were a posture dual-task that required a subject to rapidly and accurately touch a sensitive pod of the BlazePod system while standing on a foam surface over a force plate. Reaction time, number of accurate hits of the pod tapping, and center of pressure (COP) of bilateral stance were recorded. Twenty minutes of treadmill walking was conducted between the pre-test and post-test. HRV during treadmill walking was only analyzed in the central 10 min, when treadmill speed had reached 5 km/h. The elliptical COP area represents the size of postural sway in the posture dual-task. Leg blood flow was restricted in the blood flow restriction (BFR) condition by applying a blood pressure cuff (100 mmHg) to the most proximal portions of both legs. The control (NBFR) condition was treadmill walking with no blood flow restriction. During treadmill walking, heart rate was monitored

The study consisted of pre-test and post-test measurements, along with two separate sessions of BFR treadmill walking and NBFR treadmill walking conducted 1 month apart (Fig. 1). Each participant completed both experimental protocols, BFR walking and NBFR walking. The allocation of participants to the two protocols followed the balanced order principle, with fourteen participants first undergoing BFR walking and then NBFR walking and the remaining participants undergoing the same experiments in the reverse order.

After a 5-min rest, each participant's heart rate was measured at rest for 3 min. Participants then performed three 60-s posture dual-task trials, with a 3-min break between each trial, during both the pre-test and post-test. The posture dual-task involved the concurrent tasks of tapping light-pods and maintaining a bilateral static stance on a foam surface. For the light-pod tapping task, three LED pods were arranged in a triangular shape at eye level on a wall, 20 cm in front of the subjects. The LED pods emitted five colors: purple, red, yellow, blue, and green. During each run of the tapping task, only one sensor light was set to purple, red, or yellow, while the other two were blue or green. Participants were instructed to ignore the blue and green lights and to tap the target sensor light as quickly as possible during a 1.5-s light-on period, each of which was followed by a 0.5-s light-off period. Failure to respond within this time frame was considered a missed target. The next tapping run began 1.5 s after the completion of the previous run. Each posture dual-task trial contained 30 turns of light-pod tapping. Cortical activities during the posture dual-task were recorded with scalp EEG.

Between the pre-test and post-test, participants were instructed to walk on a treadmill for 20 min under the BFR (lower limb blood flow restriction combined with treadmill walking) and NBFR (treadmill walking without blood flow restriction) conditions. The initial speed was set at 2 km/h and progressively increased by 0.5 km/h every 30 s until a final walking speed of 5.0 km/h was reached. Participants then walked on the treadmill at 5.0 km/h for 14 min. Afterward, the treadmill speed was progressively decreased by 0.5 km/h every 30 s until the end of the treadmill walking session. Heart rate variations were monitored throughout the treadmill walking. In the BFR condition, participants wore inflated pneumatic cuffs (SAGA Fitness Inc., Australia) on the proximal portions of both thighs with an occlusion pressure of 100 mmHg. Higher occlusion pressures, such as 200 mmHg, were not considered because they can lead to an increased cardiovascular response and unnecessary discomfort, especially in the aged population [23]. In the NBFR condition, participants did not receive any vascular constraint during treadmill walking. After completion of the treadmill walking, the pneumatic cuffs were removed. Post-test measurements were initiated after a 5-min rest period following the treadmill walking session.

### Instrumentation setting

The heart rate was recorded using a Polar H10 HR sensor (Polar Electro Ltd., Taiwan), which held against the skin by an adjustable elastic-polymer strap to detect the electrical signals of the heart (ECG). The trajectory of the center-of-pressure (COP) during the posture dual-task was measured with a force plate (Kistler Type 9260A, Switzerland) and an amplifier (DAQ for BioWare Type 5695B, Switzerland). The COP data were digitized in BioWare software (Type 2812A, Switzerland). The light-pod tapping sequence was pre-defined by the BlazePod™ system (Play Coyotta Ltd, Israel) with high test–retest reliability in response time. During the posture dual-task, EEG of 34 channels were recorded using a NuAmps amplifier (NeuroScan Inc., EI Paso, USA) and Ag–AgCl scalp electrodes, following the International 10–20 system. Reference electrodes were placed on each side of the mastoid process (A1/A2). Electrooculography (EOG) data were collected with electrodes placed at the outer canthus of the left and right eyes to subtract eye movement and blink artifacts. The impedance of all electrodes was below 5 kΩ, and they were referenced to linked mastoids on both sides. The EEG and COP systems were synchronized by the AD controller of the LabView platform (LabView v.8.5, National Instruments, USA), with a sampling rate of 1 kHz.

### Data analysis

Posture performance of the dual-task was represented by the 95% confidence ellipse sway area of the COP trajectory (Fig. 1). The performance of the concurrent light-pod tapping of the posture dual-task was represented by the average reaction time (in milliseconds) of correct taps and the total number of correct taps within a trial. The mean heart rate (HR_mean_) and natural logarithm of the root mean square of successive differences (Ln-RMSSD) of the pre-test, post-test, and during treadmill walking were determined based on RR intervals with exclusion of differences more than 20% compared to the preceding RR intervals. HR_mean_ and Ln-RMSSD during treadmill walking were analyzed for the middle 10 min of the session (6th to 15th second), when the walking speed had reached a stable 5 km/h. Ln-RMSSD is a reliable index of activity of the parasympathetic (vagal) branch of the autonomic nervous system [32]. Heart rate data were analyzed in Kubios heart rate variability software (version 3.5.0, Kubios Oy, Kuopio, Finland).

The EEG data of an experimental trial were filtered between 4 and 35 Hz using a zero-phase finite impulse response (FIR) filter. This study did not analyze EEG data in the delta and gamma bands, as they are susceptible to movement artifacts and muscle activity of the neck. Eye blinks in the EEG were corrected with the NeuroScan 4.3 software program (NeuroScan Inc., EI Paso, TX, USA), based on a bipolar vertical EOG channel. Then the conditioned EEG data of part of the run were segmented into 2-s epochs [10, 11]. For the regional activity of the EEG, the spectral power in each epoch was calculated using Fast Fourier Transformation (FFT) with a frequency resolution of 0.2 Hz [9]. The power spectra of all channels were pooled across epochs to determine the spectral peaks of each EEG channel in the sub-bands (theta (4–7 Hz), alpha (8–12 Hz), and beta (13–35 Hz)) in an experimental trial. The regions of interest for various sub-bands were denoted on the EEG channels, and regions where the sub-band peak amplitudes were significantly different between the BFR and NBFR conditions (*p* < 0.05) were examined with paired t statistics. The spectral peaks of the electrodes in the regions of interest of the three trials were averaged in the BFR and NBFR conditions. Inter-regional connectivity of brain activities was represented with the phase-lag index (PLI) calculated from EEG data of 30 electrode pairs across all sub-bands. The PLI described the distribution asymmetry of phase differences in the instantaneous phases of two time-series based on the Hilbert transformation. If $\varphi (t)$ is the phase difference, the PLI is defined thus: $PLI=\left|E\left\{sgn(\Delta \varphi (t))\right\}\right|$, where *sgn* is a function that extracts the sign of a real number. The PLI measure offers the advantage of minimizing bias from common sources such as volume conduction [46]. The adjacent matrix A ∈ R^n×n^ representing the PLI connectivity between all EEG channels, where n denotes the number of nodes in the matrix. The PLI-based functional connectivity was calculated with the HERMES function in Matlab [35].

**Fig. 2:**
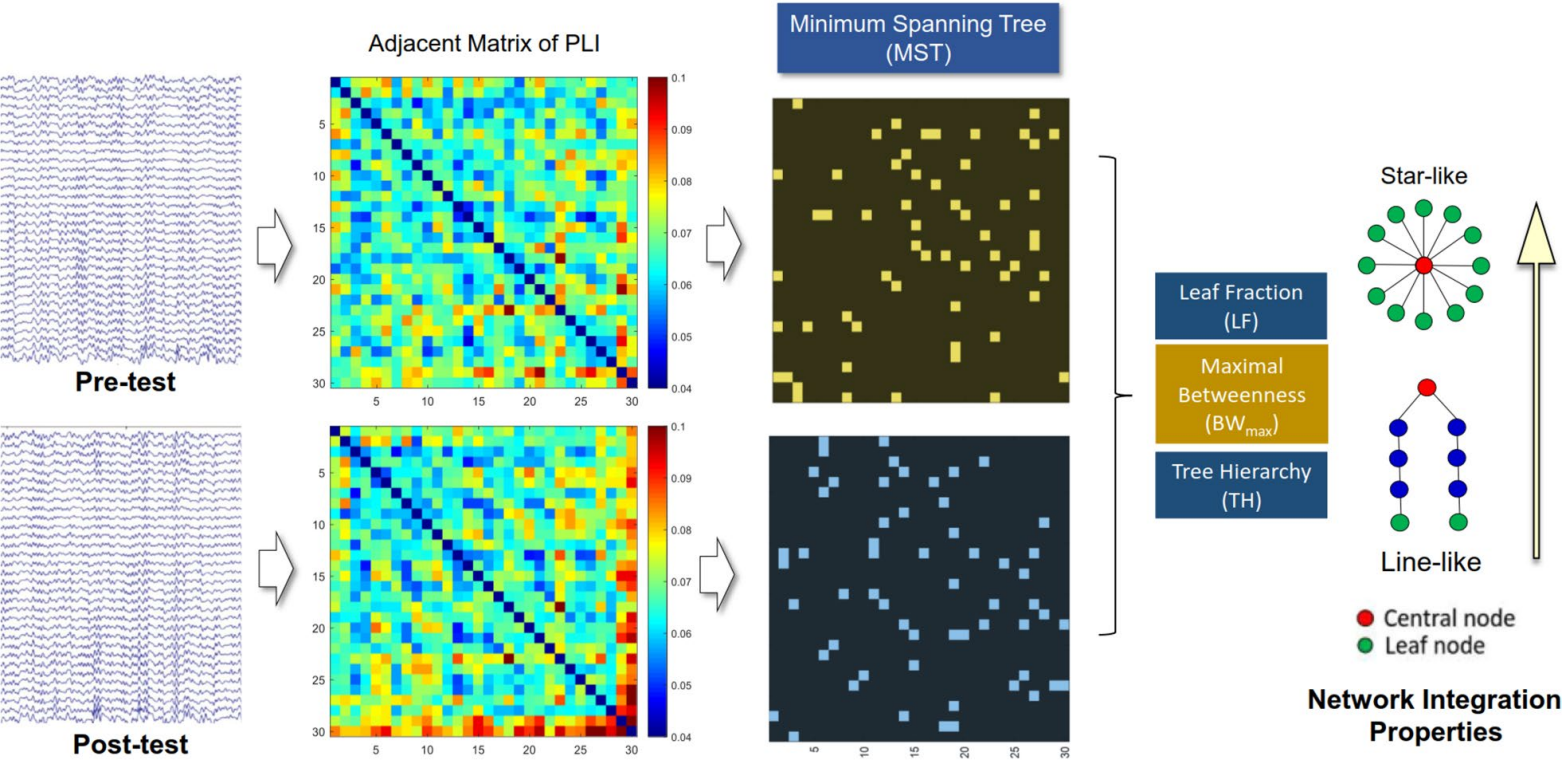
A schematic illustration of the contrast minimum spanning tree (MST) network between the pre-test and post-test conditions. MST network is constructed with adjacent matrix of phase lag index (PLI) of electrode pairs. Network integration properties are characterized with MST variables (leaf fraction, maximal betweenness (BW_max_), and tree hierarchy (TH))

For each EEG sub-band epoch and each participant, a minimum spanning tree (MST) subgraph was constructed using a PLI adjacency matrix (Fig. 2) [10, 11]. The MST represents a simplified network structure where high-probability connections between all nodes are retained without forming loops through the shortest paths [47]. The tree topology of the MST was quantified using three parameters: leaf fraction (LF), maximal betweenness (BW_max_), and tree hierarchy (TH) [11] (Fig. 2). Leaf fraction represents the ratio of nodes with only one edge. BW_max_ is defined as the highest value of betweenness centrality in the network, a measure based on shortest paths. TH characterizes the hypothesized optimal topology, promoting efficient organization while preventing information overload of central nodes. An MST with higher LF, BW_max_, or TH close to 0.5 tends to have a star-like topology, while an MST with low LF, BW_max_, and TH close to 0 tends to have a line-like topology. The former network is more efficient and integrated, as information flow from all nodes converges to fewer central hubs. MST and network properties were calculated using functions in the Brain Connectivity Toolbox [39].

### Statistical analysis

The data are expressed as mean ± standard deviation. A paired t test was used to compare normalized differences (ND) ((post-test-pre-test)/pre-test) in dual-task variables and heart rate variables between the BFR and NBFR conditions. For EEG variables, paired t test was used to contrast differences in the pooled spectral peaks of various sub-bands of the EEG channels in the regions of interest between the BFR and NBFR conditions. Hotelling's T^2^ statistics were used to contrast the MST variables (LF, BW_max_, and TH) of the different sub-bands between pre-test and post-test for the BFR and NBFR treadmill walking. The statistical significance level was defined as *p* < 0.05. All statistical analyses were conducted in IBM SPSS software (version 19.0; IBM Corp, USA).

## Results

### BFR-related changes in task performance of the posture dual-task

In the light-pod tapping task, both groups displayed decreasing trends in reaction time (RT) and increasing trends in the number of accurate hits. The normalized decrease in RT was more pronounced in the BFR condition (− 10.44% ± 4.64%) than in the NBFR condition (− 5.61% ± 5.74%) (*p* = 0.002) (Table 1A). Additionally, the normalized increase in the number of accurate hits showed a marginal difference between the BFR condition (8.70% ± 5.28%) and NBFR condition (6.09% ± 3.93%) (*p* = 0.067) (Table 1A). Regarding the posture task, the ND in the COP area was significantly smaller in the BFR condition (− 11.04% ± 21.96%) than in the NBFR condition (8.25% ± 38.42%) (*p* = 0.012) (Table 1B).

**Table 1:** Means and standard deviations of posture dual-task performance for the NBFR and BFR conditions

(A)					
Light-pod tapping task		Pre-test	Post-test	ND (%)	Statistics
RT (ms)	NBFR	1058.1 ± 131.1	995.9 ± 112.7	− 5.61 ± 5.74%	*t*_*26*_ = − 3.450, *p* = 0.002
	BFR	1063.3 ± 164.9	948.2 ± 125.8	− 10.44 ± 4.64%^**^	
Accurate Hits	NBFR	66.9 ± 4.8	70.8 ± 4.1	6.09 ± 3.93%	*t*_*29*_ = 1.914, *p* = 0.067
	BFR	68.4 ± 6.0	74.1 ± 4.4	8.70 ± 5.28%^§^	

(B)					
Posture task		Pre-test	Post-test	ND (%)	Statistics
COP Area (cm^2^)	NBFR	20.25 ± 11.20	21.96 ± 15.69	8.25 ± 38.42%	*t*_*26*_ = 2.684, *p* = 0.012
	BFR	22.20 ± 13.14	20.34 ± 16.08	− 11.04 ± 21.96%^*^	

This table also summarizes the results of paired t-tests, contrasting the normalized differences (ND) in posture dual-task performance between the NBFR and BFR conditions. The performance of the light-pod tapping task was measured by reaction time (RT) and the number of accurate hits of the flashing light. The performance of the posture task was assessed by the elliptical area of the center-of-pressure (COP) trajectory. (^**^: BFR < NBFR,* p* < 0.005; ^*^: BFR < NBFR,* p* < 0.05; ^§^: BFR > NBFR, *p* < 0.07) (BFR: blood flow restriction; NBFR: non-BFR)

### BFR-dependent HR variables during treadmill walking

HR_mean_ during treadmill walking was significantly lower in the BFR condition (100.1 ± 12.3 beats/minute) than in the NBFR condition (105.9 ± 14.2 beats/minute) (*p* < 0.001) (Table 2A). The BFR condition also exhibited higher Ln-RMSS (*p* = 0.038) than did the NBFR condition during treadmill walking. Table 2B presents the HR_mean_ and Ln-RMSSD values for the pre-test and post-test measurements in both the NBFR and BFR conditions. The results of paired t-tests revealed that the normalized increase in HR_mean_ was significantly smaller in the BFR condition (9.05 ± 17.04%) than in the NBFR condition (21.17 ± 20.25%) (*p* = 0.017). Conversely, the normalized increase in Ln-RMSSD was significantly greater in the BFR condition (18.89 ± 17.59%) than in the NBFR condition (1.72 ± 23.61%) (*p* = 0.002).

**Table 2:** Means and standard deviations of mean heart rate (HR_mean_) and natural logarithm of root mean square of the successive differences in successive heart beats (Ln-RMSSD) for the NBFR and BFR groups during treadmill walking (A), in the pre-test and post-test (B)

(A)			
	NBFR	BFR	Statistics
HR_mean_ (beat/min)	105.0 ± 14.2	100.1 ± 12.3^*^	*t*_*26*_ = − 4.124, *p* < 0.001
Ln-RMSSD (ms)	2.56 ± 0.49	2.80 ± 0.45^†^	*t*_*26*_ = 2.186, *p* = 0.038

(B)					
		Pre-test	Post-test	ND (%)	Statistics
HR_mean_ (beat/min)	NBFR	76.5 ± 9.8	92.2 ± 14.1	21.17 ± 20.50%	*t*_*26*_ = − 2.548, *p* = 0.017
	BFR	76.4 ± 8.6	83.1 ± 15.2	9.05 ± 17.04%^*^	
Ln-RMSSD (ms)	NBFR	2.75 ± 0.46	2.73 ± 0.36	1.72 ± 23.61%	*t*_*26*_ = 3.485, *p* = 0.002
	BFR	2.77 ± 0.50	3.21 ± 0.35	18.89 ± 17.59%^††^	

In (B), this table summarizes the results of paired t-tests, contrasting the normalized differences (ND) in HRmean and Ln-RMSSD between the NBFR and BFR conditions. (*: BFR < NBFR, p < 0.05; †: BFR > NBFR, p < 0.05, ††: BFR > NBFR, p < 0.005)

### BFR-related variations in local activity and inter-regional connectivity

**Fig. 3:**
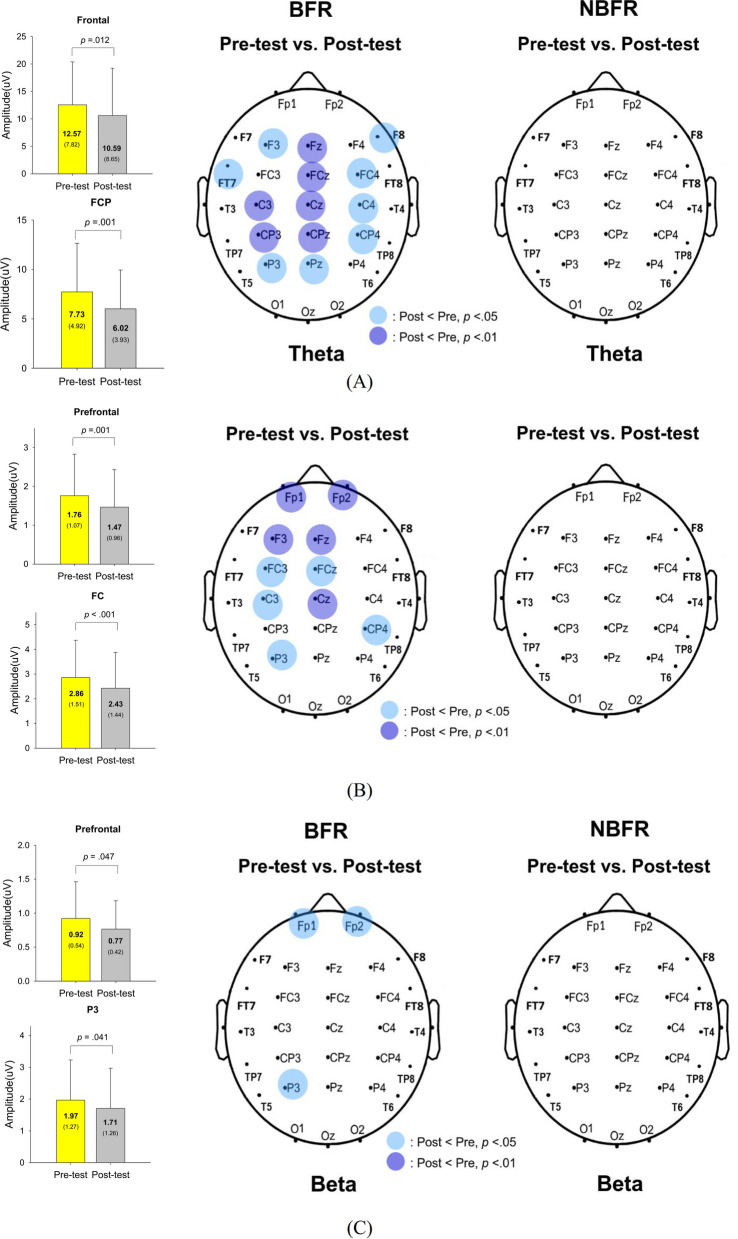
A comparison of pooled topological spectral mapping of scalp EEG in the theta (4–7 Hz), alpha (8–12 Hz) and beta (13–35 Hz) bands between the pre-test and post-test for the BFR and NBFR conditions. The dark and light blue circles represent the regions with lower spectral peaks in the post-test than in the pre-test, with statistical significance at *p* < 0.01 and *p* < 0.05, respectively. In **A**, in the BFR condition, the mean amplitudes of the theta spectral peaks in the frontal (F3 and Fz) and fronto-centro-parietal (FCP) areas (FCz, FC4, C3, Cz, C4, CP3, CPz, CP4, P3, and Pz) were smaller in the post-test than in the pre-test. In **B**, in the BFR condition, the prefrontal and target fronto-central areas showed smaller alpha spectral peaks in the post-test. In **C**, in the BFR condition, the mean amplitudes of the beta spectral peaks in the prefrontal and P3 electrodes were smaller in the post-test than in the pre-test

In the BFR condition, the regional theta spectral peak in the frontal (F3 and Fz) and fronto-centro-parietal (FCP) areas (FCz, FC4, C3, C4, Cz, CP3, CP4, CPz, P3, and Pz) was significantly smaller in the post-test than in the pre-test (*p* < 0.05) (Fig. 3A). Specifically, the mean amplitudes of the theta spectral peaks in the frontal (*p* = 0.012) and FCP areas (*p* = 0.001) were notably reduced in the post-test. However, no significant change in theta spectral peaks was observed in the NBFR condition (*p* > 0.05). For the alpha spectral peaks, regional activity in the prefrontal (Fp1 and Fp2) and fronto-central areas (F3, Fz, FC3, FCz, C3, and Cz), CP4, and P3 exhibited significant decreases in the post-test in the BFR condition (*p* < 0.05) (Fig. 3B). Furthermore, the mean amplitudes of the alpha spectral peaks in the prefrontal (*p* = 0.001) and fronto-central (*p* < 0.001) areas were significantly smaller in the post-test than in the pre-test. Conversely, no significant change in the alpha spectral peak was found in the NBFR condition (*p* > 0.05). Regarding the beta spectral peak, a global decrease in mean amplitude was observed in the prefrontal (Fp1 and Fp2) (*p* = 0.047) and P3 (*p* = 0.041) areas (Fig. 3C) in the BFR condition, while no significant change in the beta spectral peak was noted in the NBFR condition (*p* > 0.05).

**Fig. 4:**
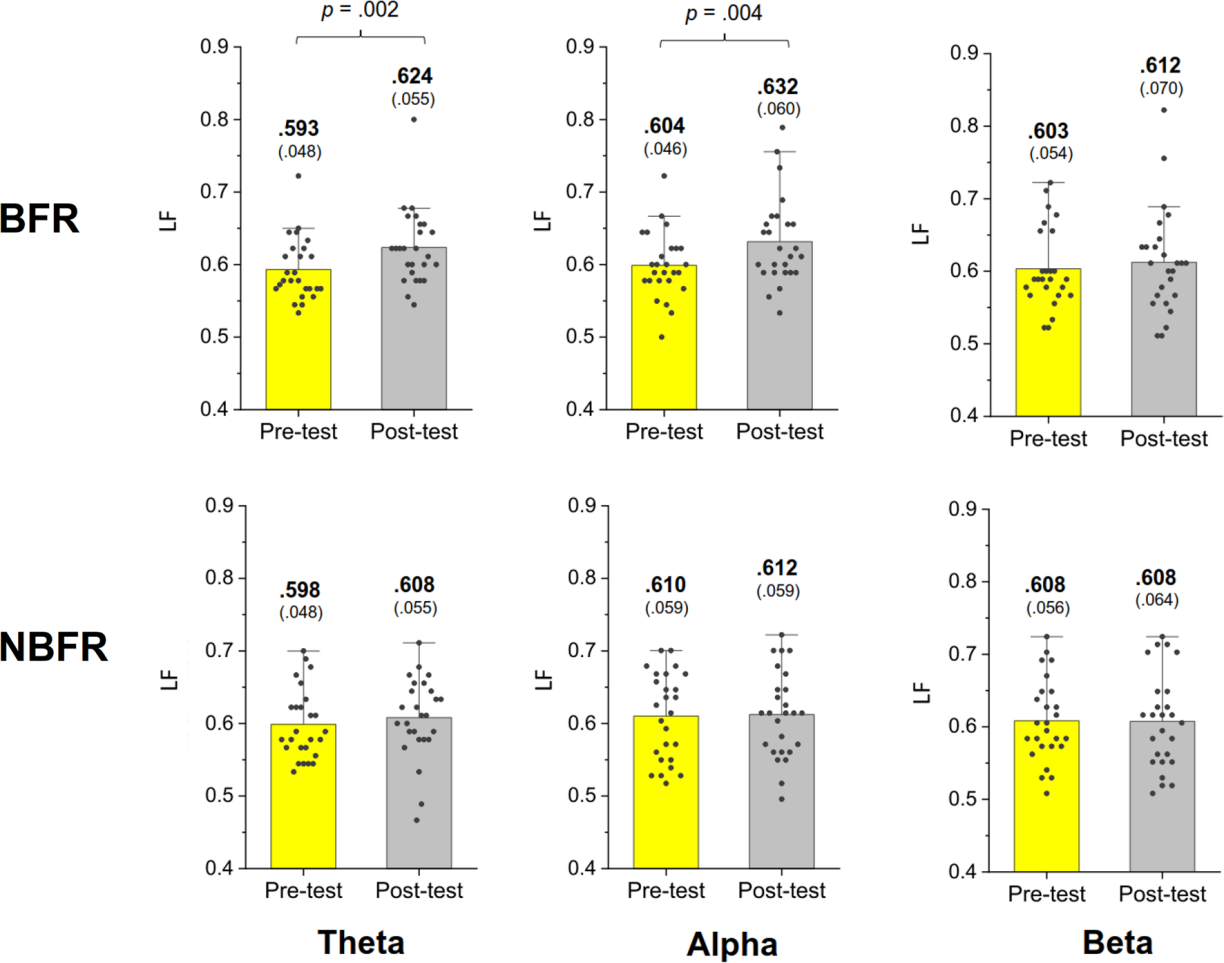
The comparison of leaf fraction of MST of the theta (4–7 Hz), alpha (8–12 Hz), and beta (13–35 Hz) bands between the pre-test and post-test in the BFR and NBFR conditions

**Fig. 5:**
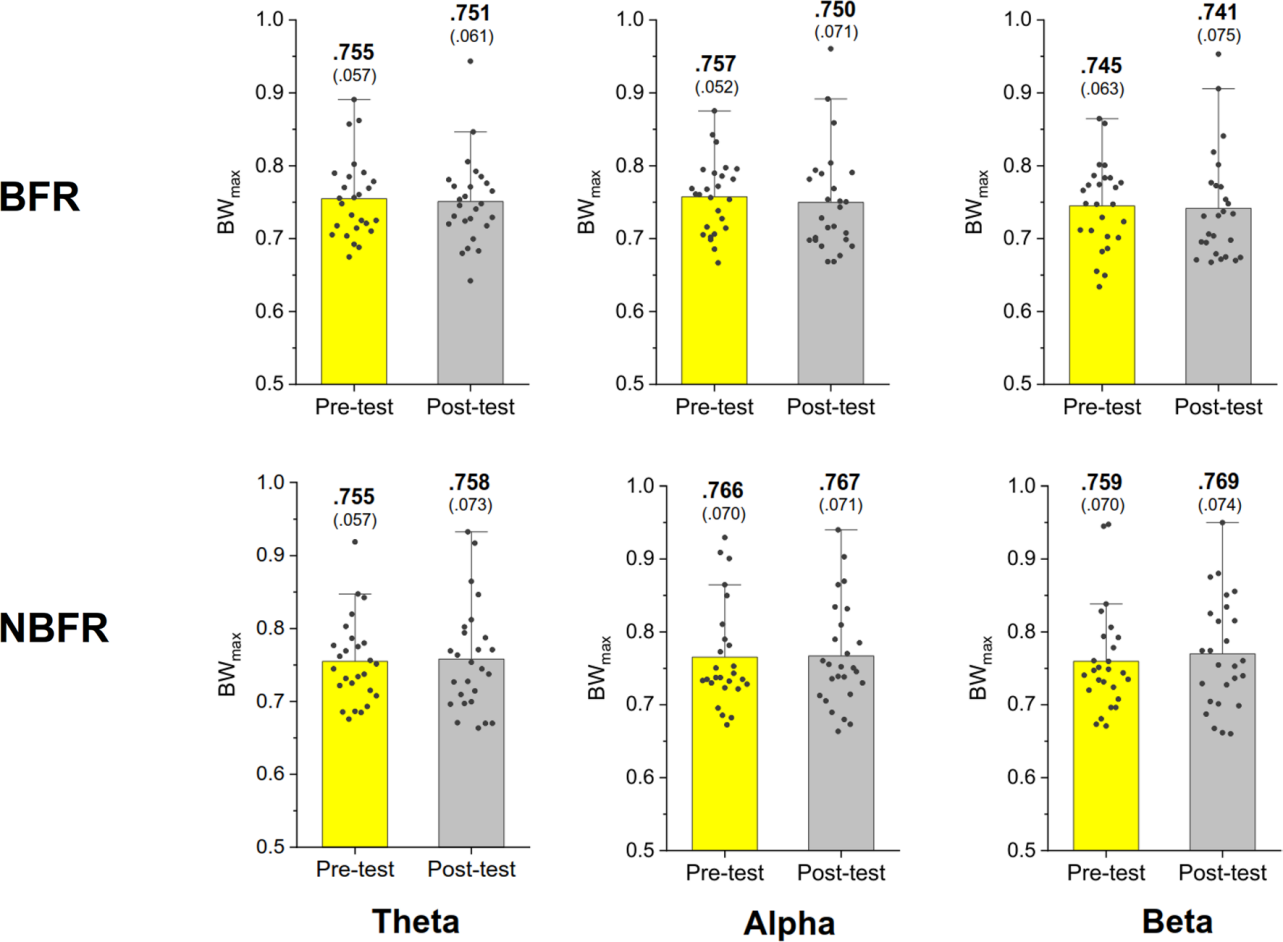
The comparison of maximal betweenness (BW_max_) of MST of the theta (4–7 Hz), alpha (8–12 Hz), and beta (13–35 Hz) bands between the pre-test and post-test in the BFR and NBFR conditions

**Fig. 6:**
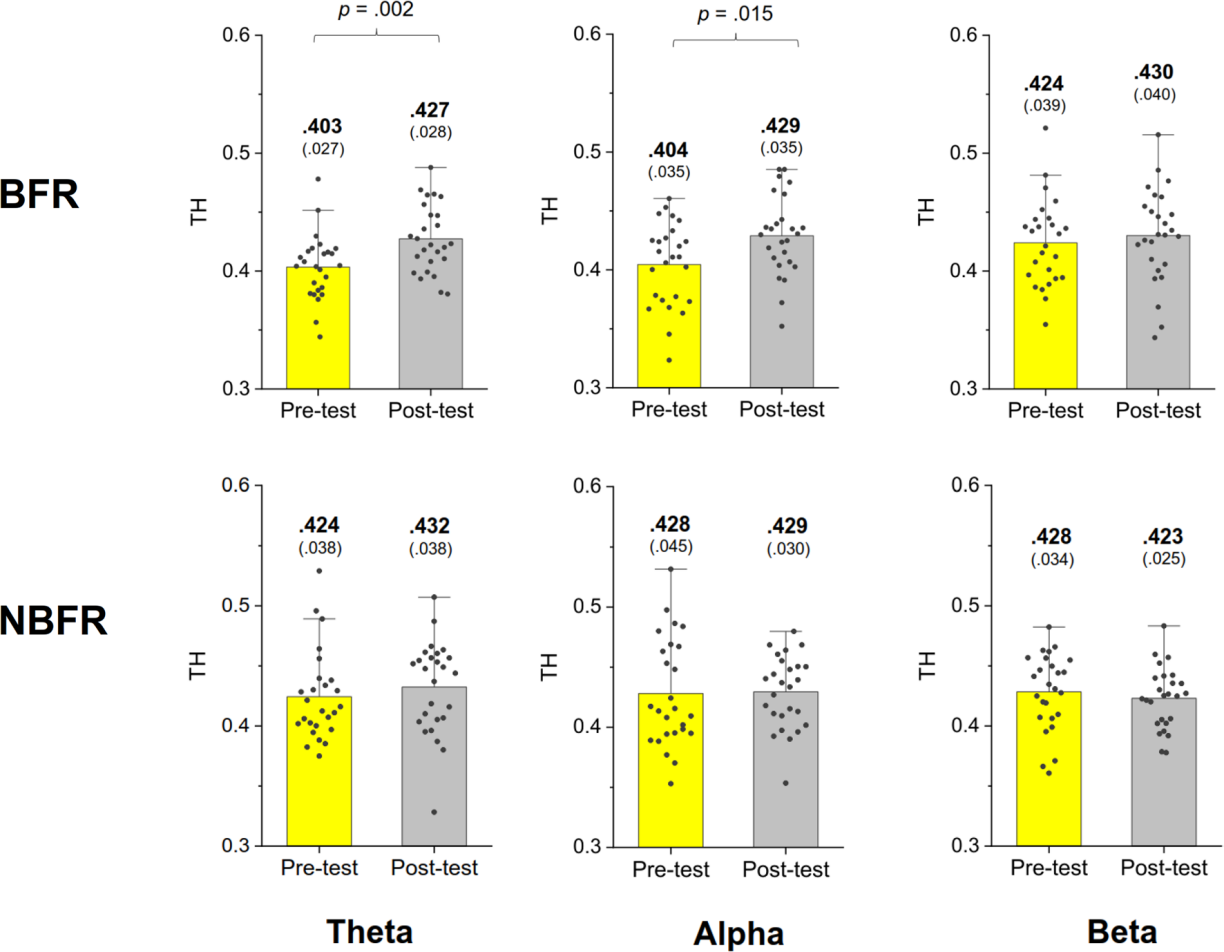
The comparison of tree hierarchy (TH) of MST of the theta (4–7 Hz), alpha (8–12 Hz), and beta (13–35 Hz) bands between the pre-test and post-test in the BFR and NBFR conditions

In the BFR condition, the results of Hotelling's T^2^ statistic revealed a significant difference in LF of the MST networks between the pre-test and post-test (Wilks' Λ = 0.523, *p* = 0.002) (Fig. 4, top row). In contrast, there was no significant difference in LF between the pre-test and post-test in the NBFR condition (Wilks' Λ = 0.954, *p* = 0.778). Further post-hoc tests indicated that in the BFR condition, LF of the theta (*p* = 0.002) and alpha (*p* = 0.004) bands was higher in the post-test than in the pre-test (Fig. 4, top row). Analysis with Hotelling's T^2^ statistics revealed no significant difference in BW_max_ between the pre-test and post-test for either the BFR (Wilks' Λ = 0.970, *p* = 0.867) or NBFR condition (Wilks' Λ = 0.971, *p* = 0.875) (Fig. 5). TH in the pre-test significantly differed from that in the post-test in the BFR condition (Wilks' Λ = 0.652, *p* = 0.018) (Fig. 6, top row), whereas there was no significant difference in the NBFR condition (Wilks' Λ = 0.945, *p* = 0.720). Further post-hoc tests indicated that post-test TH was higher than pre-test TH in the theta (*p* = 0.002) and alpha (*p* = 0.015) bands in the BFR condition (Fig. 6, bottom row).

## Discussion

This study discovered that low-occlusion BFR treadmill walking improved posture dual-task performance in older adults as compared with regular treadmill walking. BFR treadmill walking lowered the heart rate and increased heart rate variability. The dual-task benefits due to BFR treadmill walking are associated with favorable reductions of local brain activity in the theta, alpha, and beta bands and enhanced network integration in the theta and alpha bands.

### Performance improvement of posture dual-task after BFR treadmill walking with low occlusion pressure

Because of the decline in attentional resources due to prefrontal degeneration, older adults often adopt a safer "posture prioritization strategy" to stabilize posture, at the cost of a less-important secondary task [37]. Interestingly, the older adults in the present study exhibited enhanced dual-task performance after engaging in BFR treadmill walking (Table 1A, B). Our findings align with recent studies that have reported cognitive enhancements, such as improvements in attention, executive functioning, and memory, resulting from aerobic exercise combined with BFR after short-term or long-term interventions [25, 48, 51]. The primary distinctions between the present study and previous research are the utilization of lower occlusion pressure (100 mmHg) in the BFR walking protocol, despite the controversy about the effect of cuff pressure on cognition. Unlike previous studies that applied a final occlusion pressure of 200 mmHg to both legs, our study employed a much lower occlusion pressure of 100 mmHg, yet we still observed comparable cognitive benefits. It is known that high occlusion pressure could lead to leg discomfort and increased cardiovascular responses resulting from stimulation of the sympathetic system [33]. In practice, the use of lower occlusion pressure in the BFR protocol not only helps maintain adherence to walking but also reduces cardiovascular stress in the elderly [13]. Studies using high occlusion pressures have linked cognitive improvements after weeks of BFR training to the release of neurochemical substances such as BDNF and lactate [52]. However, the mechanisms of BFR treadmill walking may differ depending on the occlusion pressure (low vs. high) and intervention duration (short-term vs. long-term). The immediate neurophysiological mechanisms underlying the dual-task improvement after BFR walking remain unclear.

### Vagal modulation of HRV and posture dual-task with low pressure BFR

Given the increases in Ln-RMSSD during treadmill walking with BFR and in the post-test (Table 2A and B), it is plausible to suggest that the improvement in dual-task performance could be linked to enhanced vagal modulation. The priming effect on hemodynamic response and sympathetic-vagal balance after the combination of aerobic exercise and BFR appears to be pressure-dependent [27]. Low-intensity resistance exercise with BFR at 60% and 80% of systolic pressure has been reported to result in better parasympathetic activity, with significant reductions in blood pressure and enhanced heart rate variability [16, 24]. In contrast, high-load exercise or BFR with high pressure could favor sympathetic predominance as a consequence of discomfort [41], which counterbalances the vagal effect of BFR modulation due to NO release from the endothelium [21].

Vagal modulation could lead to lasting aftereffects that improve task performance in older adults. Lower heart rate variability in older adults often corresponds to worse performance on global cognition tests [19]. The functional linkage between task performance and heart rate variability is rooted in the prefrontal–vagal network, a bidirectional communication pathway linking the prefrontal cortex and the nucleus tractus solitarius in the brainstem for vagal regulation. Individuals who regularly engage in exercise training tend to have higher heart rate variability and enhanced prefrontal efficiency, allowing them to sustain attention more effectively and allocate attentional resources more flexibly during dual-task performance [14, 15]. Notably, a priming effect that enhances vagal activity has been observed via stimulation of the respiratory vagal nerve after moderate-to-high aerobic exercise for 20–30 min in healthy adults [7] and clinical populations [42]. In this context, BFR treadmill walking with low occlusion pressure may induce an acute priming effect on dual-task facilitation similar to that of moderate-to-high aerobic exercise.

### BFR-related cortical reorganization for posture dual-task improvement

Compared to their younger counterparts, older adults demonstrate heightened reliance on the cerebral cortex during dual-task scenarios, especially in terms of compensatory prefrontal activation [12, 20]. Interestingly, the older adults in the present study showed reduced regional EEG activity in the dual-task after BFR treadmill walking (Fig. 3A–C). The reductions in theta power observed in the frontal lobe and fronto-parietal areas (Fig. 3A) were consistent with previous findings showing that cognitive difficulty or task interference decreased during multitasking [3, 5]. This observation may indicate that the older adults detected errors or conflicts, and they did not have to engage in corrective actions to maintain dual-task performance. More accurate light-pod tapping was also helpful in reducing the prefrontal beta activity observed in the post-test dual-task in the BFR condition (Fig. 3C). In this context, the exertion of higher inhibitory control by lateral prefrontal areas is not necessary for executive control adjustments [28] or for conveying more information to optimize task performance [17]. These adaptive changes in regional brain activities with improved dual-task quality did not occur following NBFR treadmill walking. However, alpha suppression in the attentional network, including the frontal and sensorimotor areas, in the post-test dual-task after BFR walking (Fig. 3B) may reflect enhanced attention and alertness to task-relevant cues, as commonly observed following moderate-intensity exercise [18, 30]. The effect is pertinent to the recovery phase after parasympathetic activation, which may contribute to an enhancement of sustained attention and flexible utilization of brain reserves in older adults to meet the demands of dual-tasking.

MST connectome analysis was used to characterize the topological differences in major neural communication routes before and after BFR treadmill walking (Figs. 4 and 6). In the post-test, the higher leaf fraction and tree hierarchy in the theta and alpha bands suggested a high degree of network integration shifting towards star-like centralized organization [44]. In the context of increasing leaf fraction and tree hierarchy, the development of the MST network from childhood to adolescence also suggests a shift in network configuration from a line-like structure to a more star-like one [44, 47]. The MST network tends to remain star-like in adults and gradually transitions to a more line-like topology with age in later life. The star-like MST network indicates more efficient information transformation within the backbone network. A loss of network efficiency leads to a shift from a star-like MST network toward a more decentralized line-like organization in subhealth populations and patients with cortical dysfunction, such as smokers (Su et al. 2017), Alzheimer's disease [8], and others. Hence, the observed reorganization of MST networks may pertain to their roles in coping with task switching and interference control [40]. Shaw et al. [43] found that increased postural challenge enhanced connectivity in the fronto-centro-temporo-parietal theta network, while the inhibition of alpha networking between fronto-centro-temporo-parietal and parieto-occipital regions decreased with higher demands of the secondary cognitive task.

## Conclusion

Within the context of the heart–brain axis, this study is the first to reveal vagally-mediated improvement in posture dual-task performance in older adults following a single bout of walking with leg BFR using low occlusion pressure. Leg BFR increased heart rate variability and lowered the mean heart rate during treadmill walking and in the post-test. The dual-task benefits observed in the posture dual-task following BFR treadmill walking were characterized by decreases in regional cortical activities across bands, along with an increase in network integration. These plasticity changes induced by BFR contributed to the vagally-mediated neural economy, which were observed along with dual-task improvements. In practice, combined treadmill walking with low-pressure BFR holds promise for combating cognition-relevant injurious falls in at-risk older adults. 

## Data Availability

Data cannot be shared as participants were informed that their data would be stored confidentially, in accordance with the rules of the local ethics committee. Code to generate the EEG metrics is available under request.

## Abbreviations

BFR: Blood flow restriction
BNDF: Brain-derived neurotrophic factor
BW_max_: Maximal betweenness
COP: Center-of-pressure
ECG: Electrocardiography
EEG: Electroencephalography
FCP: Fronto-centro-parietal
HR_mean_: Mean heart rate
EOG: Electrooculography
FIR: Finite impulse response
FFT: Fast Fourier Transformation
LED: Light-emitting diode
Ln-RMSSD: Natural logarithm of the root mean square of successive differences
LF: Leaf fraction
MST: Minimum spanning tree
ND: Normalized differences
NO: Nitric oxide
NBFR: Non-blood flow restriction
PLI: Phase-lag index
RT: Reaction time
RR: R-wave peak to R-wave peak
TH: Tree hierarchy
